# Health policy – why research it and how: health political science

**DOI:** 10.1186/1478-4505-12-55

**Published:** 2014-09-23

**Authors:** Evelyne de Leeuw, Carole Clavier, Eric Breton

**Affiliations:** Public Health, La Trobe University, Melbourne, VIC 3000 Australia; Département de Science Politique, Université du Québec à Montréal, Case postale 8888, succ. Centre-Ville, Montréal, Québec H3C 3P8 Canada; Ecole des Hautes Études en santé Publique (EHESP), Avenue du Professeur Léon-Bernard - CS 74312, 35043 Rennes cedex, France

**Keywords:** Juggling, Policy, Politics, Theory

## Abstract

The establishment of policy is key to the implementation of actions for health. We review the nature of policy and the definition and directions of health policy. In doing so, we explicitly cast a health political science gaze on setting parameters for researching policy change for health. A brief overview of core theories of the policy process for health promotion is presented, and illustrated with empirical evidence.

The key arguments are that (a) policy is not an intervention, but drives intervention development and implementation; (b) understanding policy processes and their pertinent theories is pivotal for the potential to influence policy change; (c) those theories and associated empirical work need to recognise the wicked, multi-level, and incremental nature of elements in the process; and, therefore, (d) the public health, health promotion, and education research toolbox should more explicitly embrace health political science insights.

The rigorous application of insights from and theories of the policy process will enhance our understanding of not just how, but also why health policy is structured and implemented the way it is.

## Background: policy is not an intervention

Systems perspectives on population health development entered research and practice agendas from the early 1980s. Two complementary traditions emerged; McLeroy et al. [1] consider health behaviour change as the resultant of the complex interaction between behavioural determinants and higher-level environmental and policy conditions. The Ottawa Charter for Health Promotion [2] emphasises the development of supportive environments, reorientation of health services, and building of health public policy to enable societies making healthier choices the easier choices. Neither tradition has managed to comprehensively shift research focus, nor has it generated evidence of effectiveness from individual behaviourist perspectives to deep insight in the workings of broader social determinants of health.

Yet, the capacity to develop and assess policy processes for health promotion has been appreciated and formalized across jurisdictions. For Europe, the CompHP Core Competencies Framework for Health Promotion Handbook ([3], p. 1) states that: "*A competent workforce that has the necessary knowledge, skills and abilities in translating policy, theory and research into effective action is recognised as being critical to the future growth and development of global health promotion*". Paragraph 5.7 of the Australian Health Promotion Association’s Core Competencies for Health Promotion Practitioners [4] states that "*an entry level health promotion practitioner is able to demonstrate knowledge of: health promotion strategies to promote health—health education, advocacy, lobbying, media campaigns, community development processes, policy development, legislation*". Interestingly, the most detailed listing of policy competencies is provided by the US National Commission for Health Education Credentialing under section ‘7.5 Influence Policy to Promote Health’ [5], as indicated below.

*7.5.1 Use evaluation and research findings in policy analysis;*

*7.5.2 Identify the significance and implications of health policy for individuals, groups, and communities;*

*7.5.3 Advocate for health-related policies, regulations, laws, or rules;*

*7.5.4 Use evidence-based research to develop policies to promote health;*

*7.5.5 Employ policy and media advocacy techniques to influence decision-makers.*

Yet, for many health educators and health promoters ‘policy’ is a critical yet elusive concept [6]. On the one hand, they recognise public policy as a critical element in shaping the opportunities for the profession and setting the parameters for its effectiveness [7]. On the other, they consider policy as an abstract construct best left to politicians, or as a distal determinant of health that can be changed following Cartesian heuristics. Those that have attempted the latter and have failed would claim that policy-making is not just abstract but obscure, without any appreciable logic.

Within the health promotion and health education realm the discourse around policy has been obfuscated further by lumping policy change together with ‘environmental’ perspectives on ‘(social) ecological’ approaches for promoting or improving health behaviour [8]. Most of the North American literature remains implicit and surprisingly limited in defining, describing, or operationalising what such policy change is or encompasses. For instance, Kahn-Marshall and Gallant [9] carried out a meta-analysis to assess whether there is demonstrable effect of environmental and policy change on workplace health. However, nowhere in the piece they operationalise what precisely constitutes ‘policy change’ (or for that matter, ‘environmental change’) – it appears to be some undefined notion of modification in organisational parameters.

In this paper, we contend that public health experts, health educators, and health promoters would benefit from considering public policy through the lens of political science rather than through the lens of intervention research. The key arguments are (a) that policy is not an intervention, but drives intervention development and implementation; (b) that understanding policy processes and their pertinent theories is pivotal for the potential to influence policy change; (c) that those theories and associated empirical work need to recognise the wicked, multi-level, and incremental nature of elements in the process; and, therefore, (d) that the health promotion and education research toolbox should more explicitly embrace health political science insights.

### Health, policy

Although this is not the place to fully review the academic and practice-oriented discourse around the concepts of ‘health’ or ‘policy’, it seems important to delineate a few issues around the use and application of the expression ‘health policy’.

Policy is in itself a fuzzy concept for political science scholars, variably apprehended as "*The actions of government and the intentions that determine those actions*" [10], or rather "*Anything a government chooses to do or not to do*" ([11], p. 2). Some would simply see policy as ‘The Plan’ or ‘The Law’ [6]. Richards and Smith say that "*‘Policy’ is a general term used to describe a formal decision or plan of action adopted by an actor … to achieve a particular goal… ‘Public policy’ is a more specific term applied to a formal decision or a plan of action that has been taken by, or has involved, a state organisation*" [12]. De Leeuw [13], and Breton and De Leeuw [14], follow a European tradition in political science that specifies public policy as "*the expressed intent of government to allocate resources and capacities to resolve an expressly identified issue within a certain timeframe*"*.* The latter clearly distinguishes between the policy issue, its resolution, and the tools or policy instruments that should be dedicated to attaining that resolution.

Health policy is possibly an even fuzzier term. It has been described unequivocally as "*policy that aims to impact positively on population health*" [15] and has been framed as equivalent to "*healthy public policy*" [16]. Milio [17], the first to coin the latter term, later developed a glossary in which she states that "*Healthy public policies improve the conditions under which people live: secure, safe, adequate, and sustainable livelihoods, lifestyles, and environments, including housing, education, nutrition, information exchange, child care, transportation, and necessary community and personal social and health services. Policy adequacy may be measured by its impact on population health.*" More recently, healthy public policies reincarnated as Health in All Policies [18, 19]: "*a collaborative approach to improving the health of all people by incorporating health considerations into decision-making across sectors and policy areas.*" Variations on this theme have been compiled by Rudolph et al. [19].

#### HiAP conceptualisations (Appendix, Rudolph et al., 2013) [19]

"*Health in All Policies is a collaborative approach that integrates and articulates health considerations into policy making across sectors, and at all levels, to improve the health of all communities and people.*" – Association of State and Territorial Health Officers (ASTHO).

"*Health in All Policies is a collaborative approach to improving the health of all people by incorporating health considerations into decision-making across sectors and policy areas.*" –California Health in All Policies Task Force.

"*Health in All Policies is the policy practice of including, integrating or internalizing health in other policies that shape or influence the* [Social Determinants of Health (SDoH)]*…Health in All Policies is a policy practice adopted by leaders and policy makers to integrate consideration of health, well-being and equity during the development, implementation and evaluation of policies.*" – European Observatory on Health Systems and Policies.

"*Health in All Policies is an innovative, systems change approach to the processes through which policies are created and implemented.*" – National Association of County and City Health Officials (NACCHO).

"*Health in All Policies aims to improve the health of the population through increasing the positive impacts of policy initiatives across all sectors of government and at the same time contributing to the achievement of other sectors’ core goals.*" – South Australia.

‘Health policy’ , thus, is both Healthy Public Policy and Health in All Policy, and may include public health policy and health care policy. Public health policy can be conceived either as public sector (government) policy for population health (public health policy) or any policy (including corporate and other civil society approaches) concerned with the public’s health (public health policy).

‘Health care policy’ in principle focuses on health care as the organised enterprise of curing or caring for disease, disability, and infirmity, and includes efforts at regulating and organising health care professions, pharmaceuticals, financing of the healthcare system, and access to healthcare facilities. Health care in essence is disease care [20] and at its core focuses on individual outcomes rather than population issues. This is potentially confusing as in most nation-states the healthcare system includes the public health system, although efforts have been made to separate the two, for instance in Canada with the creation of the (short-lived) Health Promotion Directorate following the publication of the Lalonde Report [21], and in Kenya with a ministerial public health and sanitation portfolio [22].

When the literature refers to ‘health policy’, it usually convolutes several of the above demarcations. Most often, the phrase ‘health policy’ will be used to talk about health care policy, i.e., when actually disease or healthcare policy is meant. Admittedly, health care policy research is already a dominant and powerful driver of developments in health political science, both in terms of the number of studies and in terms of the theoretical developments it yields. However, in its scope and impact, healthcare policy research is less interested in the politics of population health. In analysing the impact and outcome of health policy, therefore, any scholar should conscientiously delineate what s/he (a) considers ‘policy’ to be, and (b) considers as the scope of ‘health’. In this paper, we use the phrase health policy in a broader way to designate all government action to improve population health, i.e., Healthy Public Policy and Health in All Policy.

### The policy process

Studying health policy requires an understanding of its development process. This is particularly important if we want to have an impact on the direction of policy and its framed health objectives. The application of theories of the policy process would enable an appreciation of the range of stakeholders and determinants of policy choice. Mackenbach [23] recently called for the further development of a ‘political epidemiology’ identifying the causal effects of political variables (structures, processes, outputs) on population health. In fact, the political sciences have developed a powerful toolbox of theories of the policy process framing these political variables (notably the work of Sabatier [24] with recent updates by Nowlin [25] and Schlager and Weible [26]).

Some of the theories that have been tried and tested include the event-driven *Multiple Streams Theory* empirically developed by Kingdon [27]; the *Punctuated Equilibrium* framework by Baumgartner and Jones [28], in which long periods of policy stability are alternated by general shifts in policy perspectives and ambitions; the *Advocacy Coalition Framework*[29, 30] that emphasises the importance of coalition formation of camps of proponents and opponents to new policy directions; the *Policy Domains* approach coming from different perspectives on network governance [31, 32]; and *Social Movement Theory*[33] arguing that disenchanted people will join social movements in order to mobilise resources and political opportunity to change public policy to their advantage. The scope of political science theory relevant to studying public policy and public policy change is even broader [34, 35], ranging from hybrid approaches that mix these perspectives [25] or address specific processes such as coalition structuring [36].

We were keen to explore to what extent this body of theories of the policy process has made in-roads into health promotion and health education research [37]. The outcome of our systematic review was no less than disappointing: we identified 8,337 health promotion and health education research articles since the ‘healthy public policy’ rhetoric became mainstream in 1986, of which only 21 explicitly and conscientiously applied a political science theory. A systematic review of the use of ‘commonly identified policy analysis theories’ to the study of social determinants of health and health equity public policy arrived at similar results, with seven articles making use of such theories out of a total of 6,200 articles [38].

The importance of rigorous application of theory to solving social problems has been proffered by Birckmayer and Weiss in their Theory-Based Evaluation approach [39], and is a key doctrine for health promotion and health education development and evaluation [40]. The selection of an appropriate theory would provide answers to questions that ask *why* things are (not) happening beyond a mere description *that* they are (not) happening. A recent example of a policy issue that was investigated without the appropriate application of theories of the policy process was authored by Gonzalez and Glantz [41]. The authors record an extensive case study of a policy failure in The Netherlands. The country is a signatory to the Framework Convention on Tobacco Control and passed comprehensive legislation regulating all aspects of its MPOWER strategy (**M**onitor tobacco use and prevention policies; **P**rotect people from tobacco smoke; **O**ffer help to quit tobacco use; **W**arn about the dangers of tobacco; **E**nforce bans on tobacco advertising, promotion, and sponsorship; **R**aise taxes on tobacco). In its implementation, however, The Netherlands failed to comprehensively ban smoking from all public drinking holes. Gonzalez and Glantz reach the conclusion that the legislative approach was unsuccessful because of "*…poor implementation efforts and the failure to anticipate and deal with opposition to the law.*" This is hardly a profound, or useful, political insight: "*It didn’t work because it didn’t work.*"

In a theory-based policy evaluation approach the authors might have made their assumptions of the phenomenon under study explicit and subsequently selected an appropriate theoretical framework. They may have already had some ‘gut feeling’ that policy implementation was to blame for the issue and applied a political science theory that claimed to identify relations between (Mackenbach’s) policy implementation structures, processes, and outputs. This may have led to the selection of Mazmanian and Sabatier’s policy implementation framework [42] – see below. Alternatively, they might have seen implementation failure as the result of a breakdown of governance arrangements between different policy levels and sectors, and selected, for instance, Hill and Hupe’s multi-level governance perspectives [43] to explain what went wrong, where, between whom and what, and how.

**Figure 1:**
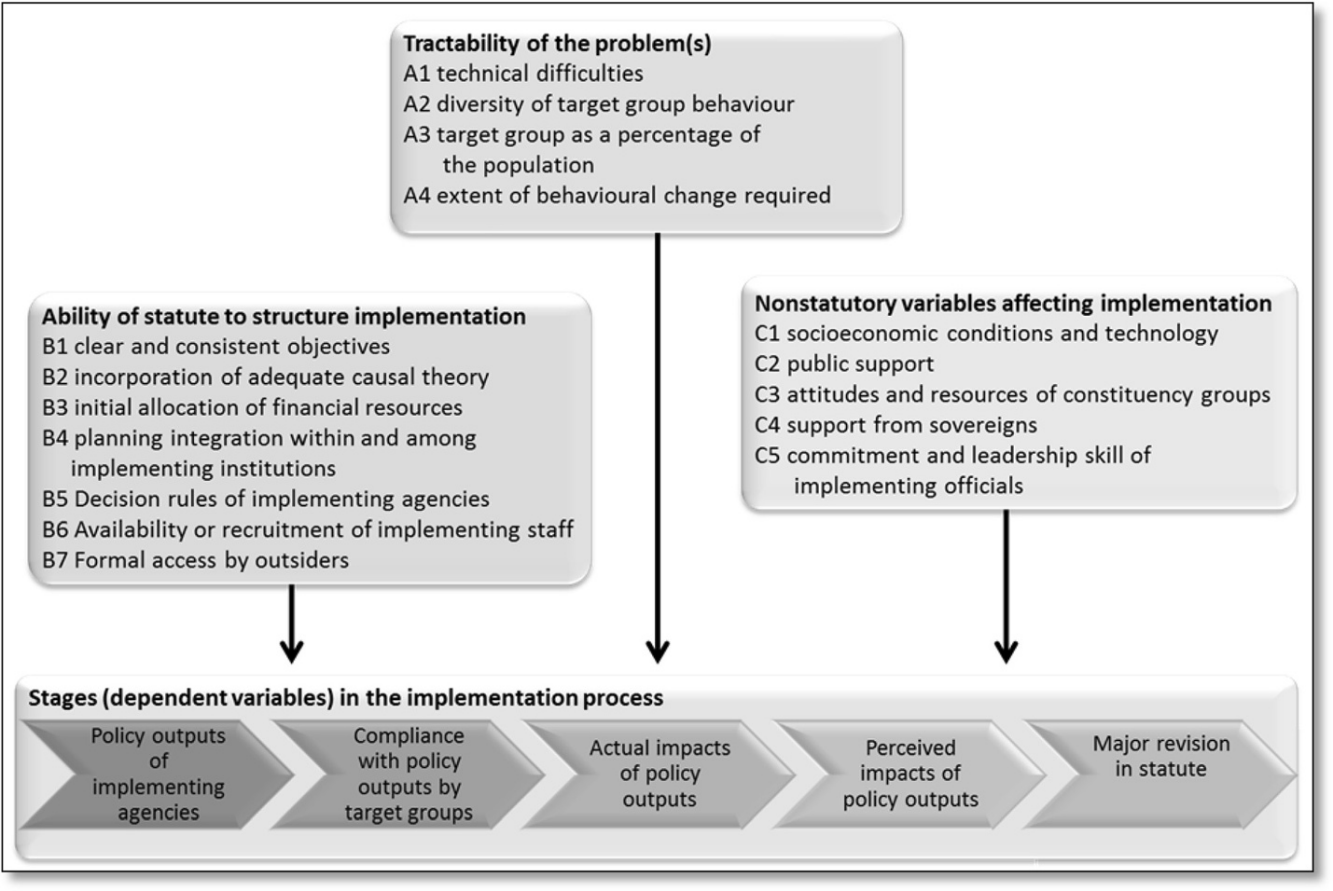
**Variables involved in the implementation process (adapted from Figure** 2 **.1 in** [42] **).**

Assuming they would have selected the Mazmanian and Sabatier model (Figure 1) [42], this would have led to the careful operationalization of variables and data to be collected – rather than drawing on a fairly randomly selected collection of informants and media expressions. The conclusions, then, would have allowed for specific propositions as regards to the identification and management of the policy problem, the ability of the Dutch governments and its agents and structures to take measures leading to implementation, and measured descriptions of facilitators and barriers beyond the control of government that impact on the implementation process. One would assume that a carefully crafted methodology in which qualitative and quantitative approaches would supplement each other would yield a much more pointed analysis and conclusions that would provide evidence-based courses of action for policy entrepreneurs and smoking-or-health activists.

A similar theoretical naïveté can be observed in a recent, albeit slightly more astute, analysis of the determinants of tobacco excise tax in the USA [44]. The analysis is more astute as the authors find that ‘political’ determinants determine tax levels. That is, the level of tax is not dependent on economic considerations, but purely on ‘political characteristics’ – these being operationalised as Democratic-Mixed-Republican control of the executive and legislative branches of State government, governor time in office, and popular attitudes toward tax levels. The conclusion is that tobacco taxes in Republican states tend to be lower, and that there are many factors (and political variables) beyond the scope of the study. Should the recommendation to the policy entrepreneur and tobacco-or-health activist therefore be to join the campaign team of the Democratic Party for the next election? The answer, as Breton and colleagues have demonstrated for the tobacco control policy development in Quebec [36], is more complicated. In their description of the evolution of advocacy coalitions (based on Sabatier and Jenkins-Smith [30] and Lemieux [45]), they show how policy elites manage and manipulate events and pool resources, and tobacco control proponents break up emerging unification of opponent coalitions. Similar policy research, with foundations in Golden, Ribisl, and Perreira data [44], would potentially highlight vastly more astute political action to solidify and secure not just tobacco control but more broadly all health policy.

### The stages heuristic and beyond

There seem to be a few barriers to the application of theories of the policy process to the health sciences in general. One is that few health scientists are trained in political science, and where they are, they do not seem to enter the health education and health promotion fields. Conversely, few students of public policy and public administration have taken an interest in health policy with the broad population and social determinant scope we described above. Most political science research is concerned with health care systems inquiry much more than with public health policy. Second, there is a lack of good benchmark studies that would set a standard for research applying theories of the policy process to public health policy, and consequently the kinds of superficial and uninsightful papers as discussed above find their way through editorial and peer-reviewed processes too easily. Third, we attribute the dearth of published studies inspired by theories of the policy process to a serious lack of (competitive) funding [14]. The proportion of grants devoted to public health is a fraction of the total medical research pool, and within the public health field funding for political research is virtually absent. Fourth, as Albert et al. demonstrated [46], members of health grant review panels do not regard social science research methods – and within that realm political science approaches – as a legitimate paradigm to study health matters. Fifth, the policy discourse in the health field is highly value-laden, intermingling debates about identity, equality [47–49], and – in the case of health care policy specifically – the role of technology and expertise [50], which clouds the legitimate application of the available evidence.

However, the two research examples given above highlight an issue that many health promotion and health education policy researchers seem to be struggling with most. This issue touches on the very nature of theories of the policy process. Theories applied in behavioural research are typically linear, at best with a feedback loop: a number of inputs (say, ‘attitudes’ and ‘beliefs’) are transformed through a number of conditioners (say, ‘social norm’ and ‘self-efficacy’) to produce intermediary (‘intention’) and final (‘behavioural’) change. In more complex behavioural systems there may be iterative and more incremental steps, and sometimes the models may take the shape of a cycle.

This, then, is also how policy development is typically modelled. Such a policy cycle can variably exist of as little as three steps (problem – solution – evaluation), four stages (agenda setting – policy formation – policy implementation – policy review) with as many as 15 sub-processes, to retrospective policy analyses that yield dozens of policy development instances, phases, and events.

All of these represent the policy process as displaying a curved linearity in which one stage –sometimes under conditions – leads to the next stage, just like the behavioural theories introduced above. While this representation of the policy process still permeates the health sciences – but also policy advice to governments [35] – policy students have now come to the realisation that policy making is a messy (some would say ‘wicked’) affair that does not neatly stick to stages.

It is not just that one stage or step coincides with another (for instance, the specification of policy alternatives may interface with the selection of policy instruments/interventions). In fact, often a step that comes ‘later’ in the stages heuristic in fact precedes an earlier phase in the cycle. A ‘real life’ example would be policy implementation. Implementation, as we have seen above, is driven by a wide array of contextual factors, including shifting power relations. Even when the policy problem is debated (as a first ‘agenda setting’ exercise), actors in the system implicitly, or by default, know that some implementation strategies will be impossible to develop. Regardless of how well-planned and analytical earlier stages in the policy process are, only certain types of interventions can be favoured. In a comprehensive review of the literature on policy instruments and interventions, Bemelmans-Videc, Rist, and Vedung formulate the ‘least coercion rule’ [51]: policy-makers choose the intervention that is least intrusive into individual choice of populations (as evidenced for obesity policy by, for instance, Allender et al. [52]). Thus, despite following the policy planning process conscientiously, the outcome in implementation terms favours communicative over facilitative or regulatory interventions. Steps in the cycle are therefore in reality rarely sequential or with feedback loops between sequential stages: often the process jumps a few steps ahead, to return to a previous step, or it finds itself going both clockwise and counter-clockwise for only sections of the cycle.

**Figure 2:**
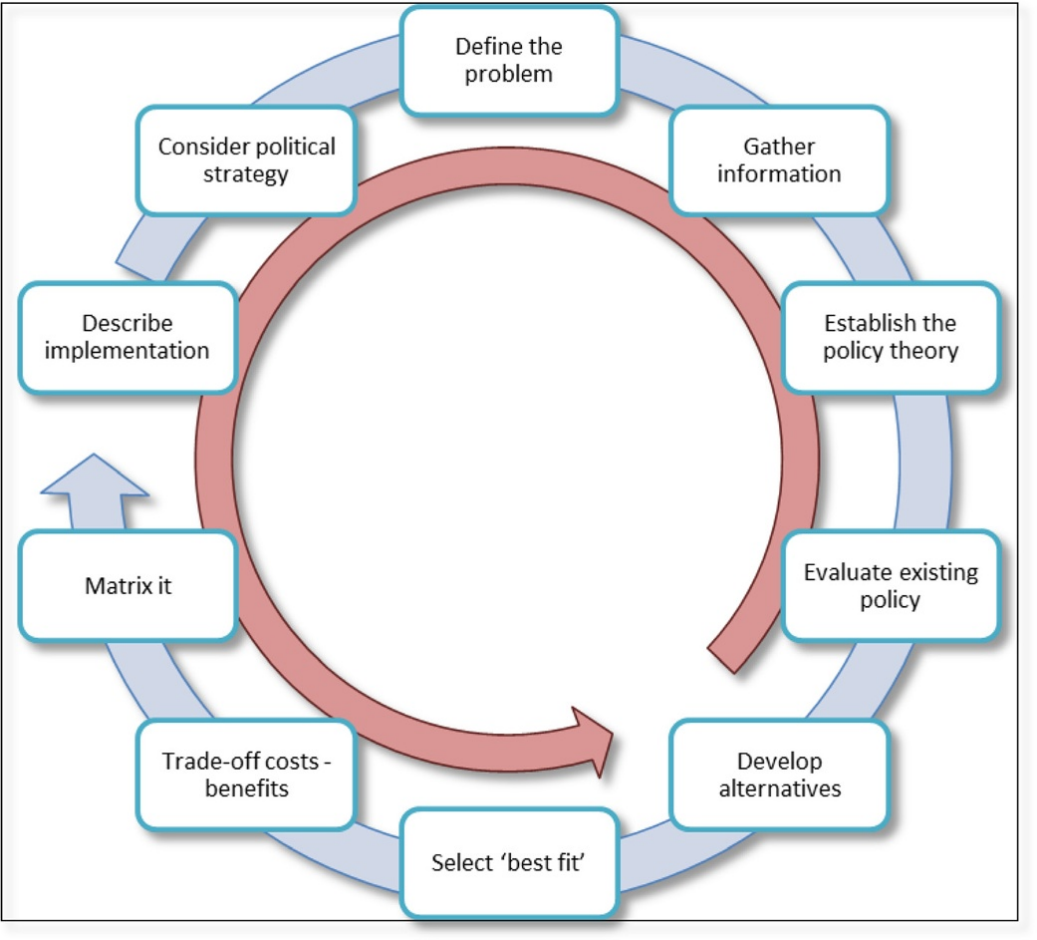
**Proposed policy process cycle for developing Health in All Policies.**

We were recently commissioned by WHO to develop a tool that would guide the development and application of Health in All Policies [53]. Through discussions with key stakeholders around the world we identified ten issues that need to be analysed and mapped in order to enhance the feasibility of Health in All Policies development. We drafted a Health in All Policies cycle (Figure 2) for discussion with Health in All Policies experts, showing both the clockwise and counter-clockwise sequential options for considering these options. The feedback on the figure demonstrated that the intuitive response to the graph was to diligently follow each of the stages, assuming there was a progressive logic to them. At the same time our panel agreed that the reality is that "*everything happens at the same time*".

This is the essence of the critique that has been voiced by political scientist on the ‘stages heuristic’ [24, 25] – that there is no causality between the different stages and therefore stages heuristic models defy theoretical testing mechanisms. The stages heuristic is useful as a mnemonic and an analytical visualisation of elements of the policy process, but does not describe the complex interactions within, between, and beyond its different features. Hassenteufel [54] furthermore argued that the analytical linearity of the stages heuristic clouds the symbolic nature of policy making in society as a sense-making activity rather than a purely methodical enterprise.

**Figure 3:**
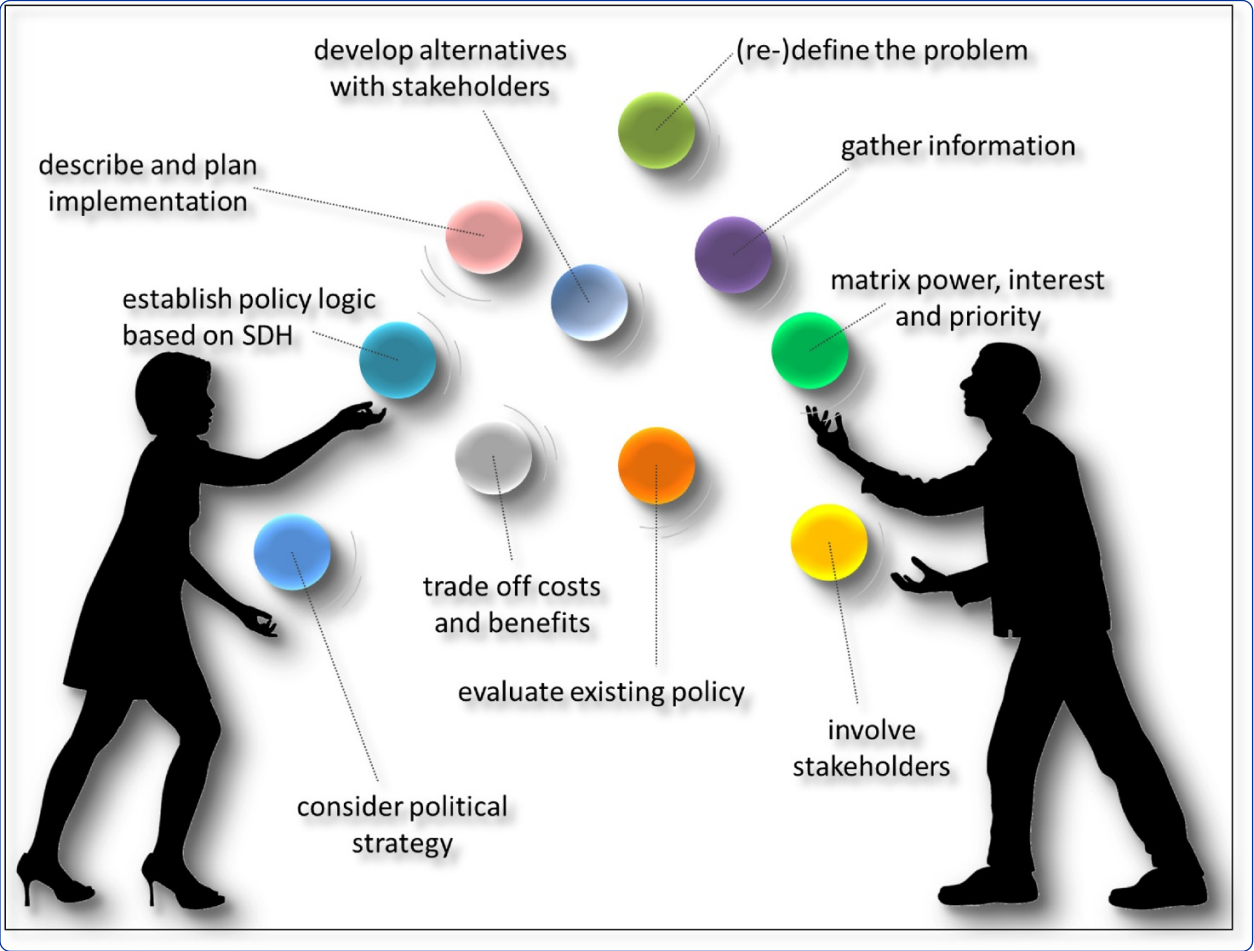
**Health in All Policies juggling process.**

We found that the best visual metaphor for this reality of the policy process is that of juggling (Figure 3).

The juggling metaphor appears to ring true to policy entrepreneurs and activists at the coal face of policy development and change. It recognises that, although keeping all balls in the air virtually simultaneously creates an apparently hugely chaotic scene, systematic and disciplined action is required at all times. Juggling is decidedly not the same as the idea of policy making as a garbage-can process (most profoundly professed by March & Olsen [55]) – the application of theories highlighted above would aim at structuring and making sense of the logic, diligence, and structure of managing a chaotic process. Theory-led discussions between academics and practitioners have been suggested to work towards this end [35]. Is the ability to keep all balls in the air also predictive of policy effectiveness?

### Assessing policy outcomes

Policies are formulated to address problems. In their ideal types, resources are allocated to develop evidence-based interventions and policy instruments and one would assume that, steeped in a validated body of knowledge, the policy will achieve its stated outcomes. However, as we have seen above, not all implementation strategies or policy ambitions are necessarily grounded in evidence. They follow the ‘least coercion rule’ [51]; are grounded in value-based rather than evidence-based policy ontologies [56]; are only symbolic to project an image of government concern [57]; or address a tangible yet insignificant element of the complexity of the real problem [58].

It is the responsibility of the policy analyst to expose such flaws through the systematic assessment of the policy process and its assumptions. Walt et al. [59] describe the multiple meanings and challenges in undertaking ‘proper’ health policy analysis. Following our argument above they contend that a conscientious, structured, and rigorous application of theories of the policy process to policy analysis is important. At the same time, however, the aims of policy analysis may be diffuse and its starting point should be to delineate its purpose. Paraphrasing a policy analysis training manual by the United Nations Environment Programme [60], the causal and final chains of drivers and consequences of policies and their contexts are hard to map, and many policies fail to include specific performance criteria or direct intervention parameters. Setting the boundaries of a policy analysis therefore becomes a negotiated process between many stakeholders, for which Pawson and Tilley [61] suggest a ‘realist’ approach that recognises the uniqueness of each policy issue and context. In showing policy ‘effectiveness’, evaluators therefore focus on intermediate policy effects rather than end-point health impact.

#### Case study: environments for health policy research – Environments for Health (E4H) policy effectiveness

In 2001, the government of the Australian State of Victoria adopted its E4H policy framework [62]. It connects with legislation that requires local governments in the State to develop Municipal Public Health Plans (MPHPs). E4H provides evidence-based guidance for the development of local policy that addresses social and environmental determinants of health in the overlapping domains of the social, built, economic, and natural environments. E4H explicitly embraces a social model of health, and the policy package provides local government with a comprehensive evidence base, capacity building for local health bureaucrats and communities, and exemplars of policy action.

Five years after adoption, the Victorian Department of Health commissioned an evaluation into E4H policy effectiveness. The evaluation objectives were to assess the extent to which the E4H Framework had:

-  Been incorporated by local governments in their policies and practices;
-  Contributed to greater consistency and quality in the scope and approach of municipal public health planning across the state;
-  Led to the integration of MPHPs with other council plans;
-  Increased the level of understanding among appropriate local government staff of the impact of the social, economic, natural, and built environments on health and wellbeing;
-  Created additional opportunities for health gain through strengthened intersectoral partnerships to address the social determinants of health; and
-  Been supported effectively by the Department of Human Services and other stakeholders [63].

The evaluation objectives were the outcome of negotiations between a range of stakeholders, including the Department of Human Services, local governments, and research sector representatives. The consequence was that hybridization of a number of political theories was required in a realist evaluation framework [61], notably policy diffusion theory [64], implementation theory [42], and Multiple Streams theory [27]. The resulting methodology drew on a range of data collection strategies:

-  Document analysis of Victorian Local Government Authorities’ MPHPs (62 plans);
-  Seventy-three individual and group interviews with key stakeholders in municipal public health planning;
-  Online survey of individuals involved in municipal public health planning (councillors, council staff, non-council organisations, and community members) (108 survey respondents);
-  Five community forums to present preliminary evaluation findings and obtain input from additional stakeholder groups.

In summary [65], the evaluation found that E4H had substantially changed the way local governments think about health; improved the way local governments plan for health; and started sectoral integration. However, developing a MPHP was frequently seen as a – statutorily required – means in itself, and implementation was often lagging. The Department of Health consequently launched programmes for implementation knowledge co-creation, capacity-building, and networking at the local level, case models for – especially economic – E4H development, and political skills.

## Conclusions

Determining the evidence of effectiveness of policy change for health is an art and a science that is still in its infancy. A systematic and theory-driven approach needs to be applied. In this paper we have demonstrated that insights from political science would allow for better and more profound insights into the reasons why and how policies fail or succeed. This is a perspective that transcends a current tradition merely describing failure or success of policy initiatives.

Our empirical material shows that policy research, assessment, and analysis needs to be a negotiated process between stakeholders that is seemingly chaotic, but in reality must be driven by the appropriate – and often hybrid – application of theories from the social sciences, notably political science.

A conscientious and transparent approach to determining what policy is and entails is a critical starting point for the further development of this field. It is recognised that such a determination is frequently impossible as even policymakers, policy entrepreneurs, and decision makers themselves are deliberately equivocal about what they pursue – the eminent economist John Maynard Keynes pointed at the need to keep options open as long as possible by writing "*There is nothing a Government hates more than to be well-informed; for it makes the process of arriving at decisions much more complicated and difficult*" [66]. It is the responsibility of public health policy analysts to expose any efforts at purposely obscuring the strictures of policy making. Good scholarly process, rigour in research, and theory-based evaluation, should enable us to do exactly that.
